# Neonatal Healthcare-Associated Conjunctivitis: A Descriptive Study from Saudi Arabia

**DOI:** 10.3390/medicina58101448

**Published:** 2022-10-13

**Authors:** Abdulaziz Alhazmi, Ismail Abuallut, Ismail Alwadani, Moayad Haddad, Bander Ageeli, Hamad Majrabi, Ibrahim Muslihi, Laila AlAli, Hana Homadi, Elaf Madkhli, Jaber Shami

**Affiliations:** 1 Faculty of Medicine, Jazan University, Jazan 45142, Saudi Arabia; 2 Medical Research Center, Jazan University, Jazan 45142, Saudi Arabia; 3 Johns Hopkins Aramco Healthcare, Dhahran 34465, Saudi Arabia; 4 King Fahad Central Hospital, Ministry of Health, Jazan 45142, Saudi Arabia

**Keywords:** healthcare-associated conjunctivitis, Jazan, Saudi Arabia, healthcare-associated infections, neonates

## Abstract

Healthcare-associated conjunctivitis (HAC) has been associated with serious ophthalmological complications in neonates, including blindness. This three-year retrospective, descriptive study was conducted between 2019 and 2021 to determine the most common bacteria associated with neonatal HAC at a tertiary-care hospital in Saudi Arabia. The inclusion criteria were defined based on the centers for disease control and prevention (CDC) guidelines for the diagnosis of neonatal HAC. When HAC was clinically suspected, conjunctival swabs were obtained from neonates and sent to the microbiology lab following standard protocols. A univariate analysis was conducted on the included samples. A total of 79 cases met our inclusion criteria and were retrospectively studied. A descriptive analysis showed that *Pseudomonas aeruginosa* was the leading cause of HAC, with 25% (20 cases), followed by *Escherichia coli* and *Klebsiella pneumonia* (11.5% for each). About 9% of the analyzed cases were positive for *Staphylococcus aureus*. Orogastric feeding was the most commonly (94%) associated factor with HAC, followed by respiratory distress syndrome (RDS) and preterm birth, which were found in 70% and 64% of the cases, respectively. To conclude, HAC is an alarming healthcare problem, and bacteria, including Gram-negative bacteria, are common causes. Thus, physician awareness, effective communication with microbiologists, and the implementation of infection control recommendations, including hand hygiene, could minimize this problem and avoid the serious complications of HAC.

## 1. Introduction

One of the most prevalent ocular diseases in newborns is conjunctivitis. It affects about 1 to 2% of neonates [1,2]. Due to its long-term sequelae and potentially serious manifestations, it is regarded as a significant public health issue [1,2]. Neonates are a vulnerable group that is at risk of developing conjunctivitis because of underdeveloped lacrimal ducts, an immature immune system, and frequent conjunctival colonization [3]. Moreover, hospitalized newborns are at a higher risk of developing conjunctivitis because of various procedures in neonatal care, such as the use of eye patches for phototherapy or contracting nosocomial infections [4]. Thus, several outbreaks of healthcare-associated conjunctivitis (HAC) have occurred as a result in neonatal units and nurseries around the globe [5,6,7,8]. Therefore, HAC stands as one of the most common healthcare-associated infections (HAI) [1].

HAC can be defined as an infection that develops after 48 h of hospitalization and is unrelated to a maternal illness [9]. Thus, a prompt diagnosis is essential to avoid complications. Gram-stain findings can be used to make a preliminary diagnosis, but conjunctival culture results should be obtained to confirm the final diagnosis of bacterial conjunctivitis [1,2]. Geographical location, prophylactic antibiotics, and particular microbial infections in healthcare facilities all affect the prevalence and range of HAC infectious pathogens. However, *Escherichia coli*, *Pseudomonas aeruginosa*, *Staphylococcus aureus*, and other Gram-positive and Gram-negative bacteria are prevalent in infections [3,4,5,6,7,8,10].

Reports from Saudi Arabia on HAC are limited, and, in this study, we aimed to identify the most common bacterial pathogens that commonly cause bacterial HAC and the factors associated with it.

## 2. Materials and Methods

### 2.1. Population and Study Design

Between January 2019 and December 2021, this retrospective study was carried out in the neonatal care units of a tertiary-care hospital in the Jazan province of Saudi Arabia. We included newborns (less than 28 days) who had conjunctivitis that was culture-proven and who had spent more than 48 h in the hospital. Infants who had any evident congenital ocular malformations or in whom two or more microorganisms had been recovered from the same sample were excluded. The patient information included demographics (gestational age, gender, and birth weight), birth history (type of birth and multiple births), length of stay in the neonatal intensive care unit (NICU), device use (mechanical days of intubation or continuous positive airway pressure, and phototherapy), sepsis, and short-term clinical outcomes. The cases of neonatal HAC were defined based on the diagnostic criteria established by the Centers for Disease Control and Prevention (CDC)/National Healthcare Safety Network that have been previously published (Table 1) [9]. Due to the absence of molecular testing at the time of this study, the studied pathogens included only culturable bacteria.

### 2.2. Microbiological Techniques

When clinically indicated, the nursing personnel at neonatal units collected eye samples with a Culturette swab (Becton Dickinson Microbiology Systems, Sparks, MD, USA). The swabs were delivered to the microbiology laboratory to be inoculated onto different growth media (including blood, chocolate, and MacConkey commercial agar plates). The plates were incubated for three days and checked every day for the presence of bacterial growth. Additionally, Gram-stained smears were prepared for an initial analysis. The traditional biochemical tests for the initial identification were used (for example, the coagulase test was used to differentiate between *Staphylococcus aureus* and *Staphylococcus* coagulase-negative, and the oxidase test was used when *Pseudomonas* spp. were suspected). Then, the automated systems MicroScan (West Sacramento, CA, USA) and Vitek 2 (bioMérieux, Durham, NC, USA) were used for the further identification or confirmation of the organisms.

### 2.3. Ethical Approval

The study was approved by the Jazan Health Ethics Committee, Ministry of Health, Saudi Arabia, with approval number #2134 (dated 28 April 2021). This study was conducted following the ethical guidelines of the Helsinki Declaration and the local guidelines of the National Committee of Bioethics, Saudi Arabia. Data were collected for clinical purposes and are available on patient charts and/or laboratory databases. The collected data were kept confidential and used for only the purpose of research within the objectives of this study. In addition, we did not include participants’ data or any other methods of identification.

### 2.4. Data Analysis

Data were tabulated and descriptive analyses were conducted, including means and frequency tables that were prepared using IBM SPSS v.23. A univariate analysis was conducted using a *t*-test and a chi-squared test.

## 3. Results

Of all the included samples (*n* = 79), females and males were equally represented (50% each), most of the newborns were delivered as single (90%) preterm (65%) deliveries, and most of these deliveries were associated with meconium (90%). Respiratory distress syndrome (RDS) was common (70%) and significantly associated with Gram-negative bacteria (85% vs. 63%, *p* = 0.046). Most mothers were multiparous (71%). An antenatal hemorrhage was observed in 9% of cases. Gestational hypertension (HTN) was present in 10% and the premature rupture of membranes (PROM) was observed in about 20% of deliveries. About half of the newborns were delivered via a normal vaginal delivery (55%). About 70% of the newborns required mechanical ventilation and food was given via an orogastric tube to 90% of the babies. A central catheter was required for 47% of the newborns, while a peripheral catheter was required for only 20% of them. Table 2 summarizes all the descriptive and univariate analyses.

Table 3 summarizes the findings regarding the types of bacteria found in a conjunctival culture based on the Gram stain and culture. About 70% of the bacteria were Gram-negative and *Pseudomonas aeruginosa* was the most common (25%), followed by *Klebsiella pneumoniae* and *E. coli* (11% each). *Staphylococcus* coagulase-negative was the most observed Gram-positive bacteria (23%), followed by *Staphylococcus aureus* (9%).

## 4. Discussion

Despite being one of the most important public health issues and a common healthcare-associated infection [1], studies on neonatal HAC are scarce in our region; thus, we aimed to describe our experience (from 2019 to 2021) in a 500-bed tertiary hospital in Saudi Arabia and report the most common bacteria associated with neonatal HAC. As there are no local criteria or protocols that define neonatal HAC, we based the diagnoses on the CDC diagnostic criteria as previously mentioned (Table 1) [9]. Given this, we found that our findings were different from what Faraz et al. reported in a central region of Saudi Arabia in 2019 when they conducted a two-year study and observed that the most common isolated bacteria were Gram-positive bacteria (60%) [11]. The picture from Yemen [12] was similar to the Faraz et al. report, where the researchers found that neonatal conjunctivitis cases were commonly caused by Gram-positive bacteria (57%). In the current study, we found that 66% of neonatal HAC cases were caused by Gram-negative bacteria, and this difference was attributed to the application of the CDC guidelines to define neonatal HAC [9]. Our findings are in line with various reports from India [8], Turkey [7], and Portugal [13] (Table 4). The findings from these studies reported 60% to 70% of cases as having a Gram-negative bacterial etiology of HAC. Thus, we believe that in the absence of a local or regional protocol in our region to define neonatal HAC, health officials need to adapt to the current definitions of the CDC criteria [9], and future improvements could be considered accordingly based on the current prevalence of etiological agents and the profile of antibiotic resistance [3,8,14].

Among the most common causes of neonatal HAC were Gram-negative bacteria, including *Pseudomonas aeruginosa*, *Klebsiella pneumoniae*, and *Escherichia coli*, which represented 47% of all presented cases (25%, 11%, and 11%, respectively). These bacteria have been usually described as causative agents for HAI in general, and HAC specifically. This result is similar to a Turkish report, in which they observed that *Pseudomonas aeruginosa*, *Klebsiella pneumoniae*, and *Escherichia coli* were the most common causes of bacterial HAC in neonates and accounted for 60% of all reported cases [7]. Likewise, Dias et al. found that these three bacteria were responsible for about 50% of reported bacterial HAC cases in neonates [13]. Further, in India, the data were not different from what was previously mentioned, in which *Klebsiella* spp., *Escherichia coli*, and *Pseudomonas* spp. represented 60% of the reported HAC cases [8]. These findings are supported by a systematic review of the bacterial profile of ocular infections, which was reported by Teweldemedhin et al. in 2017 [15]. They concluded that *Pseudomonas* spp., *Klebsiella* spp., and *Escherichia coli* are common Gram-negative bacteria, and they are more commonly associated with nosocomial infections or possibly contracted via the maternal–fetal pathway. Thus, it is crucial to call the attention of infection control teams and directors of antibiotic stewardship programs in our region toward these bacteria; they represent the majority of bacterial HAC in neonates and can lead to serious complications, as they are known for their virulence and resistance against antibiotics [14,16,17].

RDS, antenatal hemorrhage, and oxygen needs were commonly associated factors with neonatal HAC in our study (Table 2), and they have been listed as important clinical manifestations that defined high-risk newborns, which are newborns that are very likely to develop severe acute diseases and infections, including conjunctivitis [18]. Most newborn care facilities use orogastric feeding tubes and oxygen hoods, and recent research, including that on the human microbiome, has increased the awareness of the problem of microbial feeding tube infection [19,20]. Thus, it is not surprising that orogastric feeding was commonly associated with HAC (Table 2), and this finding is consistent with others [8]. Further, oxygen hoods, RDS, and preterm birth were also reported in 77%, 70%, and 64% of our study samples, respectively. These factors have been repeatedly described with neonatal morbidity in general and HAC specifically [21,22,23].

Our study bears many limitations. In the absence of molecular testing, we failed to include other significant bacteria such as *Chlamydia* spp. or *Gonococcus* spp., or indeed other microbes, including viral and fungal pathogens. Another major limitation in this study is that we were not able to acquire information regarding antibiotic sensitivity profiles (ASP), an addition that would immensely enrich this paper. In addition, we did not follow up on the included patients; as a result, the long-term sequelae of neonatal HAC could not be concluded. Moreover, it is noteworthy that some reported organisms were difficult to confirm as a reason for the infection, or were difficult to culture (e.g., *Staphylococcus* coagulase-negative). However, we believe this report is one of the few that has been published in our region and it will give insights to neonatologists, microbiologists, ophthalmologists, and infection control specialists on one of the most common problems in neonatal care. Thus, further national studies should be conducted on a larger population with a control group that includes the ASP of reported bacteria to enhance our understanding of bacterial HAC in neonates.

## 5. Conclusions

This study evaluated the most common bacterial causes of HAC in neonates based on CDC criteria. Gram-negative bacteria were common (66%) and *Pseudomonas aeruginosa* was the most common pathogen, followed by *Escherichia coli* and *Klebsiella pneumonia* (25%, 11.5%, and 11.5% of cases, respectively). *Staphylococcus aureus* was the most commonly isolated (9%) species of the Gram-positive bacteria, which represented 34% of all cases. Future studies that include a larger population and antibiotic profiles are warranted to provide a better understanding of HAC in Saudi Arabia.

## Figures and Tables

**Table 1:** CDC Criteria for HAC [9].

#	Patient has at least one of the following signs or symptoms:
1	Pain
2	Erythema
3	Swelling of conjunctiva or around eye
And.	
#	At least 1 of the following:
1	Patient has organism(s) identified from conjunctival scraping or purulent exudate obtained from the conjunctiva or contiguous tissues
2	White blood cells and organisms seen on a Gram stain of exudate
3	Purulent exudate
4	Multinucleated giant cells seen on microscopic examination of conjunctival exudate or scrapings
5	Diagnostic single antibody titer (IgM) or 4-fold increase in paired sera (IgG) for organism
#	The following are not reported as HAC:
1	Chemical conjunctivitis caused by silver nitrate (AgNO_3_)
2	Conjunctivitis occurring as a part of another viral illness

**Table 2:** Descriptive and univariate analyses of all neonatal HAC samples (*n* = 79).

Variable	*n*	%	Gram-Positive	*n* = 27	Gram-Negative	*n* = 52	*p*-Value
Female	40	50.6%	12	44.4%	28	53.8%	0.482
Male	39	49.4%	15	55.6%	24	46.2%	
Preterm	51	64.6%	18	66.7%	33	63.5%	0.810
Term	28	35.4%	9	33.3%	19	36.5%	
Single Birth	71	89.9%	25	92.6%	46	88.5%	0.710
Twin Birth	8	10.1%	2	7.4%	6	11.5%	
Meconium	71	89.9%	23	85.2%	48	92.3%	0.435
RDS	61	77.2%	17	63.0%	44	84.6%	0.046 *
Nulliparity	23	29.1%	6	22.2%	17	32.7%	0.436
Multiparity	56	70.9%	21	77.8%	35	67.3%	
Antenatal hemorrhage	7	8.9%	5	18.5%	2	3.8%	0.043 *
Gestational HTN	8	10.1%	2	7.4%	6	11.5%	0.709
PROM	15	19.0%	5	18.5%	19	36.5%	0.560
Antenatal steroid	17	21.5%	5	18.5%	12	23.1%	0.776
Vaginal delivery	35	55.7%	14	51.9%	21	40.4%	0.341
Cesarean section	44	44.3%	13	48.1%	31	59.6%	
Mechanical ventilation	54	68.4%	7	25.9%	11	21.2%	0.778
Orogastric food	74	93.7%	24	88.9%	50	96.2%	0.331
Oxygen hood	61	77.2%	20	74.1%	41	78.8%	0.778
Central catheter	37	46.8%	9	33.3%	28	53.8%	0.100
Peripheral catheter	17	21.5%	5	18.5%	12	23.1%	0.776
Birthweight (g) (mean ± SD)	1932 ± 988		2000 ± 877		1896 ± 1047		0.659

RDS: respiratory distress syndrome. HTN: hypertension. PROM: premature rupture of membranes. * The alpha criterion for the *p*-values was set to 0.05.

**Table 3:** Pathogenic bacteria causing neonatal HAC.

Bacteria	Frequency	Percent
Gram-negative (*n* = 52, 66%)		
*Pseudomonas aeruginosa*	20	25.3
*Klebsiella pneumonia*	9	11.4
*Escherichia coli*	9	11.4
*Enterobacter cloacae*	5	6.3
*Acinetobacter* spp.	4	5.1
*Serratia marcescens*	2	2.5
*Stenotrophomonas maltophilia*	3	3.8
Gram-positive (*n* = 27, 34%)		
*Staphylococcus aureus*	7	8.9
*Streptococcus* spp.	2	2.5
*Staphylococcus* coagulase-negative	18	22.8

**Table 4:** Some reports from various regions in the last ten years with the prevalence of Gram-positive and Gram-negative bacterial HAC in neonates.

Study [Reference]	Year	Study Period	Country	Gram-Positive Bacteria		Gram-Negative Bacteria	
				*n*	%	*n*	%
Al-rosi et al. [12]	2022	February–October 2021	Yemen	59	57	46	43
Faraz et al. [11]	2019	2016–2018	Saudi Arabia	81	60	53	40
Degirmencioglu et al. [7]	2017	2010–2013	Turkey	18	19	80	81
Goel et al. [8]	2016	2010–2011	India	8	40	12	60
Dias et al. [13]	2013	2009–2011	Portugal	8	14	60	86
Current study	2022	2018–2020	Saudi Arabia	27	34	52	66

## Data Availability

Data are available from the corresponding author upon reasonable request.
